# Cost-effectiveness analysis of newborn screening by tandem mass spectrometry in Shenzhen, China: value and affordability of new screening technology

**DOI:** 10.1186/s12913-022-08394-4

**Published:** 2022-08-15

**Authors:** Mingren Yu, Juan Xu, Xiaohong Song, Jiayue Du

**Affiliations:** 1 School of Medicine and Health Management, Tongji Medical College of Huazhong University of Science and Technology, Wuhan, China; 2 Hubei Provincial Research Center for Health Technology Assessment, Wuhan, China; 3 Department of Family Development and Maternal and Child Health, Shenzhen Municipal Health Commission, Shenzhen, China; 4 Department of Science and Education, the Fourth Affiliated Hospital of School of Medicine, Zhejiang University, Zhejiang, China

**Keywords:** Newborn screening, Tandem mass spectrometry, Inborn errors of metabolism, Cost-effectiveness

## Abstract

**Background:**

Newborn screening (NBS) can prevent inborn errors of metabolism (IEMs), which may cause long-term disability and even death in newborns. However, in China, tandem mass spectrometry (MS/MS) screening has just started. This study aimed to assess the cost-effectiveness of NBS using MS/MS in Shenzhen under the nationally recommended program, as well as evaluate the value and affordability of introducing this new screening technology.

**Methods:**

A Markov model was built to estimate the cost and quality-adjusted life-years (QALYs) of different screening programs. We compared PKU screening using traditional immunofluorescence (IF) with the other 11 IEMs not screened and all 12 IEMs screened using MS/MS, and the programs detecting different numbers of IEMs chosen from the national recommended program were also compared. A sensitivity analysis and budget impact analysis (BIA) were performed.

**Results:**

The incremental cost-effectiveness ratio (ICER) of detecting all 12 IEMs in the national program is 277,823 RMB per QALY, below three times per capita GDP in Shenzhen. MS/MS screening in Shenzhen can be cost-effective only if at least three diseases (PKU, PCD and MMA) are covered and when the screening program covers five diseases (PKU, PCD, MMA, MSUD, IVA), the ICER closely approaches its critical threshold. The BIA indicated the implementation cost of the national program to be around 490 million RMB over 10 years and showed no difference in budget between programs detecting different numbers of IEMs.

**Conclusions:**

We conclude that the newborn screening using MS/MS in Shenzhen is cost-effective, and the budget affordable for the Shenzhen government. Two concepts for selecting the IEMs to be detected are also presented. One is to choose the most cost-effective screening programs detecting highest number of IEMs to achieve a minimal ICER. The other considers the curability and affordability of the disease as the basis of healthcare decisions to screen suitable IEMs, achieving an ICER under the threshold and close to the minimum value.

**Supplementary Information:**

The online version contains supplementary material available at 10.1186/s12913-022-08394-4.

## Background

### Inborn errors of metabolism

Inborn errors of metabolism (IEMs) are a group of diseases caused by abnormal biochemical metabolic indicators which block the metabolic pathways in our bodies [1, 2]. While most published studies have numbered IEMs in the 500–700 range [3–5], a recent article estimates the number to be more than 1015 [6], which suggests a relatively higher cumulative incidence of IEMs, compared to a simple disease. There are many discrepancies in the prevalence of IEMs reported in these studies. Donald Waters et al. [7] estimated the global birth prevalence to be 50.9 per 100,000 live births based on a systematic literature review of birth prevalence and case fatality of IEMs globally. Other research indicates prevalence between 40 per 100,000 and 125 per 100,000 [8, 9]. In China, although epidemiological statistics are absent at the country level, regional data reveals an incidence of IEMs ranging from 35.3 per 100,000 to 136.4 per 100,000 [10–12]. Notwithstanding the actual incidence, it is now clear that IEMs affect a multitude of newborns and families all over the world.

Most IEMs are curable given early detection, but without prompt recognition can give rise to long-term disability and even death [13]. Since IEMs are ingravescent, and overlap in clinical manifestations, early symptoms may not be apparent or are challenging for differential diagnosis, if any [14]. The development of newborn screening (NBS) is now a critical tool in the prevention of primary diseases. IEMs can be detected via NBS in asymptomatic patients. In other words, medical intervention can be made quickly in the early stage to control disease progression [15]. Thus, morbidity and mortality associated with IEMs can be effectively reduced [16], and life-threatening or long-term sequelae prevented [17].

### Newborn screening

Neonatal screening began in the 1970s when Dr. Robert Guthrie developed the dried blood spot (DBS) analysis to measure metabolites in the diagnosis of phenylalanine (PKU) [18]. From there, NBS gradually evolved from a relatively simple test detecting a single congenital condition to a more comprehensive and complex screening system covering over 50 different diseases [19]. Screening methods advanced with the successive application of Gas chromatography/mass spectrometry (GC/MS), liquid chromatography/mass spectrometry (LC/MS), and tandem mass spectrometry (MS/MS) in NBS. Now, MS/MS is the mainstay of NBS given its high specificity and sensitivity [9, 20]. Different conditions, from the most common disease categories to rarer diseases can be simultaneously detected using filter paper spots or directly, in biological fluids [14, 21]. Following Millington et al. [22] first putting MS/MS into practice in 1990 to identify metabolic disorders in NBS, its use became widespread across countries [23, 24]. By 2010, the MS/MS newborn screening coverage in the US had reached 100% with 20–40 diseases being detected among different states [25]. Moreover, screening rates in other countries, such as Germany, the UK and Japan, also reached 90% [26].

The number of IEMs screened varies from country to country. In the US and Canada, NBS covers the largest number, 42 IEMs [27, 28]. By contrast, other countries have set the number within the range of 20–30, for example, Australia (21 IEMs), New Zealand (23), and Japan (24) [29–31], which is similar to that set in some provinces of China, such as Taiwan (24), Zhejiang (28) and Hefei (29) [32–36]. In other provinces of Mainland China, the number of IEMs screened for is very limited, only three in Beijing [37, 38] and four in Shenzhen [38]. Additionally, screening programs in South Korea, Germany, and the UK cover less than 20 IEMs, respectively 18, 16, and 9 [31, 39, 40].

The newborn screening program in China was first launched in the 1980s [41]. Since then, significant success has been achieved, with the screening rate increasing from 2% in 1995 to 97.5% in 2017 [42]. The application of the MS/MS method to newborn screening in China began relatively recently, in 2002 [43]. A study revealed that more than 60 laboratories throughout the country had performed MS/MS analysis, and about 40 newborn screening centers had developed the MS/MS screening by 2016 [44]. In China, an increasing number of NBS have adopted using MS/MS rather than the traditional immunofluorescence (IF), which is still the primary means in Shenzhen. Thus, there is an urgent need for the Shenzhen government to implement MS/MS screening throughout the city, especially in the context of “*Newborn Screening Management Measures”*. In February 2019, the Health Commission of Guangdong province revised its *“Newborn Screening Management Measures”*, highlighting the potential application of advanced technologies, such as MS/MS, to NBS in the province.

### Economic evaluation of MS/MS newborn screening

Developments in economic evaluations of MS/MS newborn screening in different countries have aimed to provide scientific and reasoned evidence to support and improve newborn screening programs, including Canada [45], Germany [16, 46], the US [47–50], the UK [51, 52], Thailand [53] and other countries [54, 55]. Most of these analyses focus on cost-effectiveness (cost-utility analysis). A smaller proportion of the articles are cost–benefit analyses [56–58]. The vast majority of published articles, excepting a study from Thailand, clearly demonstrate that MS/MS screening in their specific country setting is likely a cost-effective healthcare intervention. However, for lack of essential data about IEMs, the number of diseases incorporated in these analyses is usually less than 10. Further, the studies' results are not comparable given the varying regions, time frames, incremental cost-effectiveness ratio (ICER) thresholds, etc. employed in studies. Nevertheless, what cannot be ignored is that economic evaluations prove MS/MS newborn screening to be economically efficient, and offer policymakers helpful scientific evidence for the benefits of NBS programs.

In China, to say nothing of Shenzhen province, any economic evaluation of MS/MS neonatal screening is yet to be carried out, except by one research group [59]. However, the focus of that published article was the whole screening program rather than a specific single disease; prioritizing different IEMs for inclusion in the regional screening program remained undiscussed. Also, as the authors of the study themselves acknowledged, its major limitation was its not applying a Markov model-based analysis to evaluate the costs and health benefits of MS/MS screening in China. The Markov model can simulate the natural progression of the IEMs, providing more rigorous results. Endeavoring to correct this situation, we have conducted this study to determine the cost-effectiveness of MS/MS screening in the social and economic context of Shenzhen based on the diseases nominated by the national program. In doing so, we take into account the government budget and explore how to select the appropriate number of IEMs for detection.

## Methods

### Selection of IEMs

To guide applicants preparing the material of registration and application for amino acid, carnitine, and succinylacetone detection reagent (MS/MS), the Center for Medical Device Evaluation of National Medical Products Administration of China (NMPA) enacted *“Guiding Principles for Amino Acid, Carnitine and Succinylacetone Detection Reagent Registration”* in 2019 [60]. The guideline, based on the criteria for disease screening set by the WHO [61], recommends screening for 12 types of IEMs that are relatively common in China and suitable for screening by MS/MS. The twelve IEMs include Phenylketonuria (PKU), Methylmalonic acidemia (MMA), Primary carnitine deficiency (PCD), Medium-chain acyl-CoA dehydrogenase deficiency (MCAD), Very long-chain acyl-CoA dehydrogenase deficiency (VLCAD), Isovaleric acidemia (IVA), Glutaric acidemia type I (GAI), Maple syrup urine disease (MSUD), Citrullinemia type II (CIT-II), Citrullinemia type I (CIT-I), Propionic acidemia (PA) and Homocystinuria (HCY). Compared with the numbers of IEMs detected in other countries, it is a conservative recommendation based on China’s actual situation, thus suiting a referable and applicable pilot program for Shenzhen.

### Perspective, discount and comparators

This study measured the inputs and outputs of MS/MS newborn screening from a societal perspective. We then estimated both cost and effectiveness to calculate the ICER of expanded screening, with the cost and effectiveness discounted at an annual rate of 3%.

For the moment, PKU, CH (congenital hypothyroidism), CAH (congenital adrenal hyperplasia) and G6PD deficiency (Glucose-6-phosphate Dehydrogenase deficiency) – the four diseases included in the current screening program – are compulsorily detected by IF independently in Shenzhen.

The expanded screening program, as nationally recommended, is “12 IEMs detected by tandem mass spectrometry (MS/MS), including PKU, MMA, PCD, MCAD, VLCAD, IVA, GA-I, MSUD, CIT-II, CIT-I, PA, HCY”. Only PKU is covered in both the current program and expanded screening. Should the expanded program be implemented, PKU will be detected by MS/MS, while CH, CAH and G6PD will still invariably be detected by IF. Using MS/MS to screen the 12 IEMs does not mean that CH, CAH and G6PD would not be screened.

Since the screening program for CH, CAH and G6PD are unchanged and independent and don’t influence each other, we didn’t take them into consideration. All in all, this study compared PKU screened using traditional immunofluorescence (IF) with the remaining 11 IEMs not screened to all 12 IEMs screened using MS/MS.

In order to determine an adequate and reasonable number of IEMs, we compared the results of economic evaluations and budget impact analyses of MS/MS screening programs with different disease combinations. First, we conducted a cost-effectiveness analysis on the program detecting every disease to obtain an ICER ranking of various diseases. Since PKU is already covered in the current NBS program, we needed to concentrate only on the other 11 IEMs. We could then finally determine 11 screening strategies according to the ranking. The first strategy includes PKU and the disease with the smallest ICER, and the second includes PKU and the top two diseases. By parity of reasoning, the 11^th^ strategy is the nationally recommended program covering all 12 diseases.

### Model structure

Screening methods in Shenzhen include immunofluorescence for the current program with only the expanded screening program using MS/MS. Positive cases, detected in both the current program and expanded program, will be compulsorily screened in the positive test. We developed a decision-tree and Markov model to conduct the cost-effectiveness analysis of expanded newborn screening using the following assumptions:

(i) A child can have only one kind of disease.
(ii) The progress of IEMs is divided into several independent Markov states (health states) according to the main sequelae. And in each cycle, a child can be in only one of the Markov states.
(iii) The states’ future distribution depends only on current events, and not on those that occurred before.

**Fig. 1:**
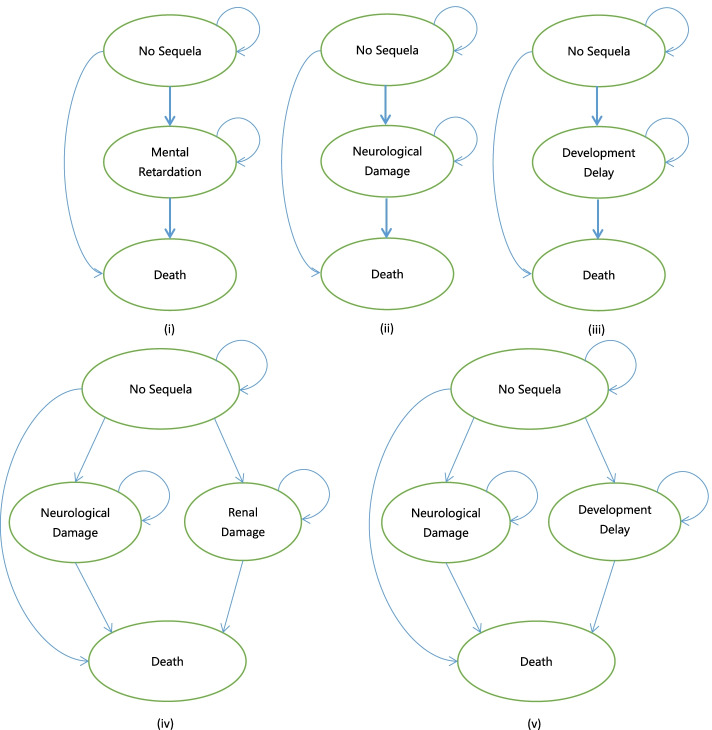
States transition of IEMs^a^. ^a^(**i**) CIT I, CIT II and HCY: Mental Retardation [49]; (**ii**) PKU, GA-I: Neurological Damage [53]; (**iii**) MCAD, PCD and VLCAD: Development Delay[47]; (**iv**) IVA, MMA and PA: Neurological Damage and Renal Damage [49]; (**v**) MSUD: Neurological Damage and Development Delay [49]

**Fig. 2:**
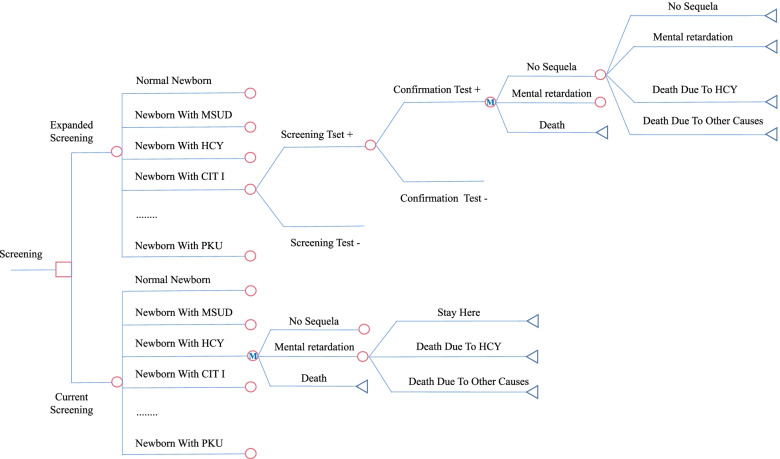
Markov model structure^a^. ^a^Compares PKU screened using IF with the remaining 11 IEMs not screened to all 12 IEMs screened using MS/MS. The square represents a decision node, circles chance nodes, and triangles terminal nodes

A half-cycle correction was made to the model. Based on the field investigation, the numbers of people participating in NBS from 2016 to 2018 in Shenzhen were 209,443, 228,206 and 206,670, respectively. Also, there were 194,393 puerperae throughout 2019, so we set the cohort at 200,000 newborns with a cycle-length of one year. The Markov model was terminated at the 82nd cycle since the average life expectancy of Shenzhen residents was 82 years in 2018. States transition of IEMs is shown in Fig. 1, and the Markov model structure in Fig. 2.

### Sensitivity and specificity

We retrieved research conducted in China and other countries and undertook a literature review to confirm the sensitivity and specificity of the two screening technologies. We assumed that, as shown in Table 1, the sensitivity and specificity of immunofluorescence are respectively 100% and 78.6%, and their counterparts are both 100%.

**Table 1:** Sensitivity and specificity of screening methods

Parameter		Mean	Reference
Fluorometry	Sensitivity	100.00%	[62]
	Specificity	78.60%	
MS/MS	Sensitivity	100.00%	[53, 63]
	Specificity	100.00%	[21, 53]

The sensitivity and specificity of MS/MS screening is 100%, meaning that the MS/MS screening accuracy is 100%, which implies that no IEM cases were missed by the MS/MS screening and similarly, no healthy cases were misdiagnosed using MS/MS. However, 100% accuracy does not imply there is no positive test. As noted earlier, positive cases detected in both the current and expanded programs, will be detected again in the positive test.

### Event probabilities

In Shenzhen, only the incidence of PKU can be collected. Therefore, this study referred to the MS/MS screening results in Zhejiang, covering 1,861,262 neonates and Nanjing, covering 850,486 neonates, as shown in Table 2. Because of the large numbers of people covered and the high maturity of widely used MS/MS, the incidence data is referable for Shenzhen.

**Table 2:** The incidence of IEMs

No	IEMs	Incidence		Reference
1	PKU	1:14,028	0.00007128	Filed investigation
2	MSUD	1:206,667	0.00000484	[33]
4	CIT I	1: 265,700	0.00000376	[33]
5	HCY	1:212,622	0.0000047	[11]
6	MMA	1:46,500	0.00002151	[34]
7	IVA	1:265,900	0.00000376	[34]
8	GA I	1:310,200	0.00000322	[34]
9	PA	1:310,200	0.00000322	[34]
10	PCD	1:23,862	0.00004191	[35]
11	MCAD	1:372,252	0.00000269	[35]
12	VLCAD	1:620,421	0.000001611	[35]

Data for the age-specific mortality and incidence of sequelae of different IEMs between the screening and non-screening groups remain scarce in China, leaving us no alternative but to refer to studies from other countries. In order to simulate the natural progress of diseases more accurately, the rate of death due to other causes was also added to the model as a parameter. This part of event probabilities is detailed in Additional file 1.

### Costs

The cost of MS/MS screening includes direct costs and indirect costs from a societal perspective. Direct costs include direct medical cost and direct non-medical cost. The former consists of expenditures for screening, confirmation, treatment (for the disease and its sequela), and follow-up, while the latter consists in family transportation costs and the program cost. Indirect costs are due to the lost productivity of families during the confirmation, treatment, and follow-up stages of NBS. Since detection in newborns occurs within six days in the hospital, families do not need to go to a specific hospital, so transportation and lost productivity costs during the screening stage are not counted.

Cost inputs used in the model are shown in Table 3. The data were derived mainly from the field investigation and estimation based on the *Guidelines for the Treatment of Rare Diseases (2019)* [64], drug prices at online pharmacies, and assumptions of this study. Also, we referred to published literature, government policies, and statistical yearbooks if parameters could not be directly obtained or estimated.

**Table 3:** Model cost parameters presented in 2018 RMB

Parameters		Mean		Reference
Screening by IF for PKU(per newborn)		23		Field investigation
Screening by IF for CH(per newborn)		23		Field investigation
Screening by IF for CAH(per newborn)		23		Field investigation
Screening by IF for G6PD(per newborn)		23		Field investigation
Screening by MS/MS (per newborn)		296		Field investigation
Confirmation by IF (per newborn)		319		Field investigation
Confirmation by MS/MS (per newborn)		296		Field investigation
Treatment for the diseases (per person-year)^a^	**Age groups**^b^	**0 ~ 5 years**	**6 ~ 82 years**	*Guidelines for the Treatment of Rare Diseases (2019)* [64]
	MSUD	13,440.90	6083.00	
	HCY	13,772.13	5952.51	
	CIT I、CIT II	48,202.46	20,833.77	
	IVA	17,567.81	13,045.01	
	GA I	17,567.81	13,045.01	
	MMA	19,023.76	24,692.53	
	PA	17,567.81	19,839.39	
	MCAD, PCD, VLCAD	15,853.26	15,853.26	
	PKU	15,000.00	15,000.00	Field investigation
Treatment for sequelae (per person-year)^c^	DD	3,064		[65]
	ND	53,400		[66]
	MR	12,000		[67]
	RD	70,213		[68]
Follow-up (per person-year)	IF (4 times)	1,276		Field investigation
	MS/MS(4 times)	1,184		Field investigation
Transportation (per year)		435		Field investigation
Lost productivity (per year)		3,060		[69]
Program cost	Sample transportation (per year)	42,000		Field investigation
	Printing (per year)	340,000		
	Cold-chain logistics (per year)	470,000		
	Software development and maintenance, equipment updating, etc (per year)	370,000		
	Utilities (per person-year)	2.52		[70], supposing 200,000 people are screened
	Administrative management (per person-year)	1.81		
	Depreciation of buildings, equipment, etc	Sunk costs		

^a^The calculation process of the treatment cost of the diseases is detailed in Additional file 2

^b^Schoen et al. [57] divide the cost of treatment into two parts at 5 years of age since, in some cases, additional care is needed for children with IEMs detected after symptoms manifest during their first five years. We also found discrepancies between different ages in the treatment and medication criteria for IEMs when referring to *Guidelines for the Treatment of Rare Diseases (2019)*. Therefore, we did the same work to estimate the treatment cost by dividing patients into two groups at age 5

^c^*DD* Development Delay, *ND* Neurological Damage, *MR* Mental Retardation, *RD* Renal Damage

### Effectiveness

Quality-adjusted life-years (QALYs) were estimated through the Markov model, multiplying the length of time in different health states by the utility value for states. We also calculated the ICER between current screening and expanded screening programs. An ICER threshold set at 568.704 RMB, three times per capita GDP in Shenzhen, was used in this study. The utility parameters of health states are listed in Table 4, estimated mainly from data in published articles. We presumed the utility of “alive state” to be 1, which means healthy.

**Table 4:** Model utility parameters

**Parameter**^a^	**Mean (range)**	**Reference**
NS	0.900(0.850–0.950)	[48]
DD	0.843(0.792–0.881)	[49]
ND	0.840(0.700–0.850)	[71]
MR	0.790(0.590–0.840)	[71]
RD	0.670(0.580–0.740)	[72]
Alive	1	Research assumption

^a^*NS* No Sequela, *DD* Development Delay, *ND* Neurological Damage, *MR* Mental Retardation, *RD* Renal Damage. The utility of the same sequela is assumed to be same across the IEMs

### Sensitivity analysis

We carried out one-way sensitivity analysis and constructed tornado diagrams to assess the uncertainty in the model and the robustness of the results. One-way sensitivity analysis in this study evaluated the influence of the discount rate in the range 0–10% (base value is 3%), with 1% as an interval of 10 categories. Tornado diagrams include factors like the incidence of IEMs, costs (e.g., the cost of screening, confirmation, transportation, etc.), and utility of health states. The incidence of IEMs was assumed to vary by 50% from their mean value, and costs were 10%. The value of utility being tested varied based on the upper/lower boundaries illustrated in published articles.

### Budget impact analysis

Implementing MS/MS screening, the expansion of diseases screened, and the increase in costs will inevitably place a burden on health expenditure, making it necessary to conduct a budget impact analysis (BIA) of MS/MS screening from the standpoint of Shenzhen’s government.

Health expenditure entailed in the frame of this study included the cost of screening, treatment, and follow-up, as well as the program cost. We assumed that these program costs remain unchanged between the expanded screening and the status quo. What should be noted is that children must receive continuous medical treatment following a positive detection. Because of the increasing numbers of patients year by year, the cost of treatment is necessarily cumulative. The treatment cost of MS/MS screening is mainly that of the IEMs without sequelae, since early detection can sharply reduce the occurrence of sequelae, as indicated in the Markov model. By comparison, the treatment cost of current screening is mainly the treatment cost for the IEMs with sequelae. Due to the high mortality rates of some IEMs, we didn’t calculate the treatment cost of patients with diseases which can cause death within their first two years.

BIA was carried out under the assumption of 200,000 newborns screened annually for ten years (2019–2028). In China, the duration of a government project, program or policy is usually five years or ten years. When we began this study, the officials of Shenzhen Municipal Health Commission claimed the budget of the expanded screening program for the next ten years. Since the starting point of this study was 2019, utilizing data from 2018, we conducted the BIA from 2019 to 2028.

Nowadays, in Shenzhen, with relevant policies and regulations released [73] and a national medical insurance system established, the cost of screening and treatment is paid for by families and the government conjointly. The government undertakes 80% of the screening cost and 60% of the treatment cost. How the rate of the medical insurance reimbursement is calculated is shown in Additional file 3.

## Results

### Cost-effectiveness analysis and BIA

#### Detecting 12 IEMs in the nationally recommended program

Table 5 shows the results of the cost-effectiveness analysis of current screening and expanded screening. The ICER is 277823RMB per QALY, below the ICER threshold (568704RMB) according to the criteria set by the World Health Organization (WHO).

**Table 5:** Cost-effectiveness analysis of expanded NBS covering 12 IEMs

**Strategy**	**E**^a^	**Incr. E**^b^	**C**^c^	**Incr. C**^d^	**Incr. C/Incr. E**	**C/E**
Expanded Screening	74.247	0.00078	478.430	217.756	277,823	6.444
Current Screening	74.246		260.674			3.511

^a^*E* Effectiveness,

^b^*Incr. E* Incremental Effectiveness,

^c^*C* cost,

^d^*Incr. C* Incremental Cost

To specify the costs of the MS/MS newborn screening program, the results of BIA are detailed in Table 6.

**Table 6:** Budget impact analysis results

**Year**	**MS/MS**^a^			**IF**^b^			**Difference**	
	**Screening cost**	**Treatment cost**	**Total sum**	**Screening cost**	**Treatment cost**	**Total sum**	**Screening cost**	**Total sum**
2019	47.36	0.27	47.63	3.68	0.58	4.26	43.68	43.37
2020	47.36	0.65	48.01	3.68	1.40	5.08	43.68	42.94
2021	47.36	1.02	48.38	3.68	2.09	5.77	43.68	42.61
2022	47.36	1.44	48.80	3.68	3.02	6.70	43.68	42.10
2023	47.36	1.77	49.13	3.68	3.74	7.42	43.68	41.71
2024	47.36	2.15	49.51	3.68	4.44	8.12	43.68	41.39
2025	47.36	2.56	49.92	3.68	5.36	9.04	43.68	40.88
2026	47.36	2.94	50.30	3.68	6.21	9.89	43.68	40.41
2027	47.36	3.27	50.63	3.68	6.90	10.58	43.68	40.05
2028	47.36	3.60	50.96	3.68	7.74	11.42	43.68	39.54
**Total sum**	473.60	19.66	493.26	36.80	41.47	78.27	436.80	415.00

^a^*MS/MS* Tandem mass spectrometry;

^b^*IF* immunofluorescence

The total cost of expanded screening is 47.63 million RMB in the first year of MS/MS implementation, increasing to 50.96 million RMB by 2028 at an average annual growth rate of 0.75%. The total health expenditure for the MS/MS screening program in Shenzhen will reach 493.26 million RMB in the next decade, costing a further 41.50 million RMB annually compared to the current NBS program. The cost of screening accounts for most of the expenditure over the whole decade (96.01%). Meanwhile, the cost of treatment increases as time goes by, accounting for only 0.56% of the total cost (0.27 million RMB) in 2019, rising to 7.06% (4.84 million RMB) by 2028.

#### Detecting some types of IEMs selected from national recommendations

The results of the cost-effectiveness analysis of screening a single disease are shown in Table 7. ICERs of all screening programs are higher than the threshold, and diseases are ranked by ICER (from minimum to maximum), i.e., PCD, MMA, MSUD, IVA, PA, MCAD, GA I, CIT I, CIT II, HCY, and VLCAD. The ICER of PCD is the minimum as 1,108,216 RMB per QALY, and the ICER of the VLCAD is maximum as 28,203,412 RMB per QALY.

**Table 7:** Cost-effectiveness analysis of screening 12 diseases singly, presented in 2018 RMB

**Diseases**	**Screening strategies**	**C**	**Incr. C**	**QALY**	**Incr. QALY**	**ICER**	**Ranking**^a^
PKU	MS/MS	410.255	203.800	74.252	0.00000	∞	
	IF	206.455		74.252			
MSUD	MS/MS	307.502	307.314	74.256	0.0001	2,939,542	**3**
	Non-screening	0.188		74.256			
CIT II	MS/MS	307.380	305.820	74.256	0.00002	18,133,638	**8**
	Non-screening	1.476		74.256			
CIT I	MS/MS	306.957	305.709	74.256	0.00001	22,672,072	**9**
	Non-screening	1.180		74.256			
HCY	MS/MS	307.007	304.463	74.256	0.00001	26,835,436	**10**
	Non-screening	2.545		74.256			
MMA	MS/MS	327.542	309.473	74.255	0.00023	1,322,605	**2**
	Non-screening	18.069		74.255			
IVA	MS/MS	308.210	305.563	74.256	0.00004	7,470,691	**4**
	Non-screening	2.647		74.256			
GA I	MS/MS	308.420	304.084	74.256	0.00002	18,017,789	**7**
	Non-screening	4.336		74.256			
PA	MS/MS	308.251	305.736	74.256	0.00004	8,728,484	**5**
	Non-screening	2.515		74.256			
PCD	MS/MS	331.625	312.330	74.254	0.00028	1,108,216	**1**
	Non-screening	19.295		74.253			
MCAD	MS/MS	306.961	305.723	74.256	0.00002	16,900,643	**6**
	Non-screening	1.238		74.256			
VLCAD	MS/MS	306.283	305.541	74.256	0.00001	28,203,412	**11**
	Non-screening	0.742		74.256			

^a^In the ranking of ICERs, the higher the rank, the bigger the ICER, indicating a less cost-effective screening program

**Fig. 3:**
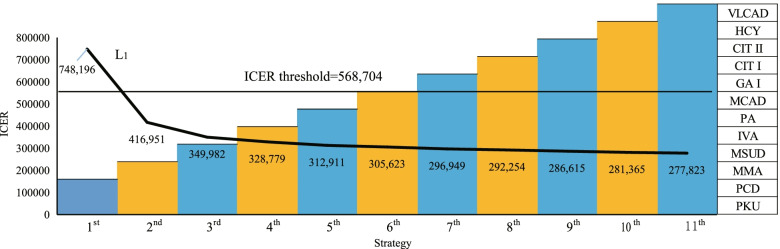
ICER of eleven screening strategies^a^. ^a^The line L_1_ in the figure illustrates the ICER of 11 screening strategies. X-axis represents the screening strategy, y-axis represents the ICER of different screening strategies and the diseases detected in strategies are showed on the right. The first strategy covers PKU and PCD, the second covers PKU, PCD and MMA. By parity of reasoning, the 11th strategy is the nationally recommended program covering all 12 diseases. Different colored columns are used to distinguish different strategies

According to the above ICER rankings of diseases, 11 screening strategies were finally examined. The ICER of each screening program is shown in Fig. 3, below. Only the ICER of the first strategy (PKU and PCD) is higher than the threshold, at 748,196 RMB per QALY. All other ICERs are below the threshold. As the number of diseases detected increases, the ICER gradually decreases and finally tends to stabilize.

**Fig. 4:**
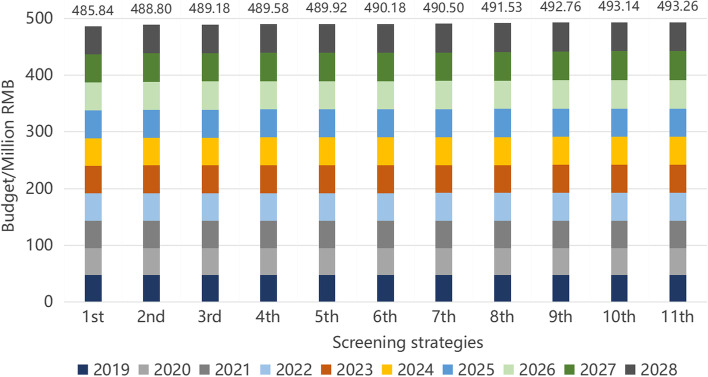
Budget impact analysis of 11 different screening strategies^a^. ^a^Columns represent the whole budget of 11 screening strategies in the decade (2019–2028). The different colored sections in one column indicate the budget for each year

The results of the BIA of the 11 screening strategies above are shown in Fig. 4. Although the screening program’s budget grows with the increasing number of diseases detected, there is no meaningful difference between single program budgets. The budget for all screening programs holds steady, near 490million RMB.

### Sensitivity analysis

**Fig. 5:**
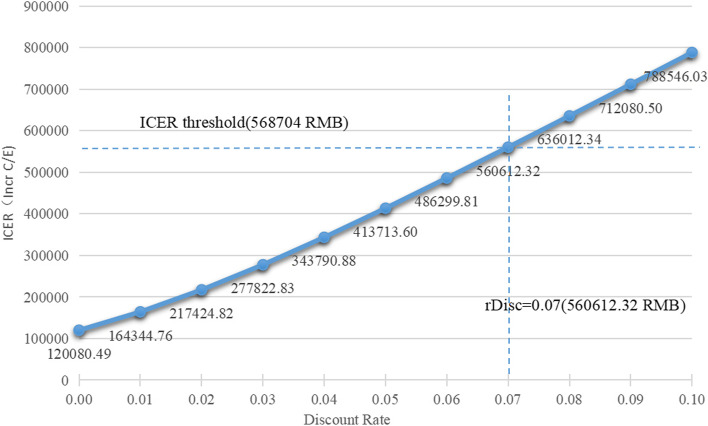
One-way sensitivity analysis of discount rate in expanded screening

**Fig. 6:**
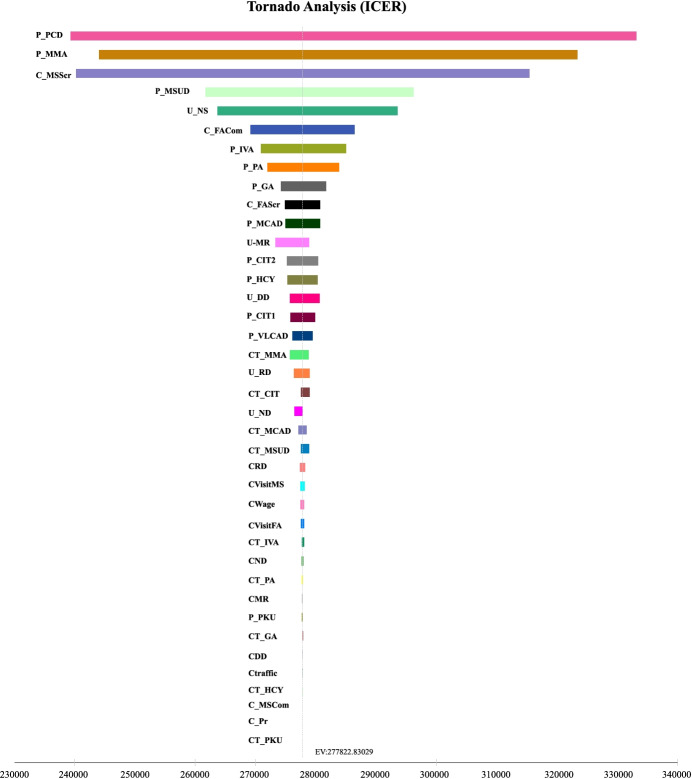
Tornado diagram of newborn screening parameters^a^, ^a^The parameters are sorted on the left according to their effect on incremental cost effectiveness ratio. The most influential parameter is on the top. EV: Expected value. P_X: The incidence of “X”, CT_X: The cost of treatment for “X”. “X” represents inborn errors of metabolism, including PCD, MMA, MSUD, IVA, PA, GA, MCAD, CIT-II (CIT 2), HCY, CIT I (CIT 1), VLCAD, PKU. U_Y: The utility of “Y”, CY: The cost of treatment for “Y”. “Y” represents states of the disease, including NS (No Sequela), DD (Development Delay), ND (Neurological Damage), MR (Mental Retardation), RD (Renal Damage). C_Pr: The program cost; C_MSScr: The cost of MS/MS screening test; C_FAScr: The cost of immunofluorescence screening test; C_FACom: The cost of MS/MS confirmation test; C_FACom: The cost of immunofluorescence confirmation test; CVisitMS: The cost of follow-up of the MS/MS screening program; CVisitFA: The cost of follow-up of the immunofluorescence screening program; CWage: The cost of lost productivity; Ctraffic: The cost of traffic

We conducted a one-way sensitivity analysis based on the nationally recommended program of 12 IEMs (Fig. 5 and Fig. 6). We discussed the discount rate and other parameters separately, since the former is remarkably influential. It turns out that the ICER is less than three times per capita GDP when the discount rate is ≤ 7% and, particularly when the discount rate is equivalent to 7%, the ICER is very close to the threshold. Also, the ICER is lower than one times per capita GDP (189,568RMB) as the discount rate is ≤ 1.5%, which means the screening program is very cost-effective.

The top three influential parameters are the incidences of PCD (P_PCD), the incidence of MMA (P_MMA), and the cost of screening by MS/MS (C_MSScr). As we can see, the incidence of diseases accounts for a large proportion of top influential parameters, and the top three are the incidences of PCD, MMA, and MSUD. Apart from disease incidence, the cost of screening by MS/MS (C_MSScr), the utility of the state without sequelae (U_NS) and the cost of confirmation by IF (C_FACom) weigh heavily for the ICER. But, no matter how parameters change within the range, the ICER always remains below the threshold, affirming that the results are robust.

## Discussion

This study considered the cost-effectiveness of MS/MS newborn screening in Shenzhen, China. From a societal perspective, we confirmed that it is cost-effective to implement the expanded screening, with some preconditions. First, it is not economically efficient to detect only one type of disease. Especially for PKU, the results illustrated IF to be a more cost-effective method currently. The factors contributing to this situation is that detecting a single disease cannot embody the advantages of MS/MS ‒ “one blood sampling for multiple diseases” [74] ‒ but reflects that MS/MS is more expensive than IF. Second, MS/MS screening can be cost-effective only if at least three diseases (PKU, PCD and MMA) are covered. The selection of IEMs for detection associates to the incidence of diseases. The higher the incidence is, the more QALYs can be saved, and the more diseases detected, and the smaller ICER of the program will be. Moreover, when the screening program covers five diseases (PKU, PCD, MMA, MSUD, IVA), the ICER closely approaches its critical value (since the ICER of the program covering six diseases accounts for more than 95% of the ICER of five diseases).

The above points can explain not only why the number of NBS programs varies from region to region but can also reveal two strategic policy ideas for selecting the number of diseases covered. One of these strategies is to screen as many IEMs as possible and get a most cost-effective screening program with a minimal ICER, like the 11th screening program mentioned above. “One blood sampling for multiple diseases” allows an MS/MS screening program to cover more IEMs without additional cost. Therefore, screening more diseases leads to more QALYs saved, making the screening program more economically efficient. The first policy idea finally can attain a minimal ICER as the decision-maker adds more and more IEMs into the NBS program, which is perhaps the reason for the US and Canada setting their number of IEMs at 42. Significantly, this strategy proposes the request to regions in terms of social, economic and medical science development. The other policy strategy is to get an almost best screening program detecting suitable IEMs, with an ICER under the cost-effectiveness threshold and close to its minimum value, like 5th screening program mentioned above. In fact, there exists the inevitable problem of how to treat these hard-to-cure diseases. Although various therapies have so far been developed [75], several factors, such as the cost of treatment, the complexity and difficulty of treatment, patient compliance, and so on [76, 77], nonetheless constitute a huge challenge for medical treatment. Without appropriate medical treatments being available, newborns detected positive can only cause families serious economic and emotional stress. It is therefore wise to include some IEMs with a high incidence and for which reasonable treatment is available in the screening program. And, in this case, the ICER of the program is already close to the minimum value. We believe that’s why some countries, such as the UK, South Korea, and Germany, have limited detectable diseases to less than 20. In brief, for the initial implementation of MS/MS screening in Shenzhen, we would suggest utilizing the second policy strategy.

In China, while economic evaluations of NBS have been made in many studies [78–84], only one has undertaken a cost-effectiveness analysis of MS/MS screening [59]. Most studies have concentrated on the traditional NBS for PKU and/or CH. The benefit–cost ratio ranges between 1:2.38 ~ 1:4.58 for PKU and between 1:3.60 ~ 1:19.94 for CH, demonstrating the economic benefit of traditional NBS. As for the results of the single study focusing on MS/MS screening, these showed the ICUR of MS/MS screening to be -768,429 RMB/QALY (MS/MS screened vs. non-screened) and the BCR was 6.09. That study’s finding of the cost effectiveness of MS/MS screening is consistent with the result of our study as well as the findings of those studies carried out in other countries [46, 48–52].

The results of this study are generally robust. One-way sensitivity analysis revealed that caution should be taken when the discount rate surpasses 7%. But, statistics indicate that China's annual inflation rate has remained at around 2% for the past decade (owing to COVID-19, the rate once approached 6%, but remained below 7%) [85]. That is to say, the implementation of MS/MS screening in Shenzhen will be cost-effective for some time to come and likely to be highly cost-effective, since inflation sometimes falls below 1.5%.

The findings of the BIA performed demonstrate that no meaningful difference exists among different programs in the whole budget. Given we choose a cost-effective screening program, in every year over the next decade budgets will all reach about 49.33 million RMB. Combining this with the ICER of a program detecting five diseases which reaches the critical value, we can conclude that if the budget of the program covering five diseases is affordable for the Shenzhen administration, it is better to screen for all twelve diseases as per the nationally recommended program. According to the *Guidelines for the Treatment of Rare Diseases (2019)*, there are specific and systematic treatments for these diseases.

Here, we also considered whether the Shenzhen government could bear the budget as proposed, and the answer is yes. According to the *Department Final Report of the Health Commission of Shenzhen in 2018* [86], the budget for public health projects in NBS (including screening for hearing, for IEMs, and for trisomies 21, 18, and 13) was 289.21 million RMB at the beginning of 2018. However, the final expenditure was 123.73 million RMB, accounting for 42.7% of the budget. This indicates the sufficiency of Shenzhen government funds to finance an expanded program. Moreover, data from *Health Statistics Summary of Shenzhen in 2019* [87] show that annual government expenditure on medical treatments and health services increased from 8.332 billion RMB in 2014 to 19.09 billion RMB in 2018, with an annual growth rate of 23.03%, which is much larger than the annual growth rate of the cost of MS/MS newborn screening program.

Another of our team’s studies[88], to investigate patients’ willingness to pay (WTP) for the MS/MS screening, revealed the average WTP value was 242 RMB, and that 68.71% (404/588) of families were willing to pay more than 200 RMB. So, the government pricing of the MS/MS screening is truly adjustable and flexible when the WTP value is compared with 20% of the screening cost (average 60.98RMB-79.57RMB per year) that a family should currently be able to afford. And if policymakers are concerned about the risks of health expenditure, it is opportune to increase the proportion of out-of-pocket payment to relieve the high-cost burdens of government. Such a policy change would, however, need to be properly considered since the aim of “*gradually providing the NBS program free of charge*” was proposed in a recent provincial policy. Briefly, the series of data presented above makes the claim that the MS/MS newborn screening program is affordable for the Shenzhen government.

Due to the lack of epidemiological investigation in China and Shenzhen, the integral local database of IEMs is still extraordinarily wanting. So, in order to solve this bottleneck problem, key recommendations are for research that outlines top priorities to enhance epidemiological studies of IEMs and advance the establishment of the IEMs database in China. Only in this way can we proceed with a cost-effectiveness analysis suiting China’s circumstances and provide further empirical evidence for the NBS program.

In this study, we assumed the sensitivity and specificity of MS/MS screening is 100%, implying that this method produces no false positive or false negative results. In fact, even if the accuracy of the technology is sufficiently high, false positives in the practical screening program can still occur. Such occurrences of false positive cases can incur additional costs, including for treatment, confirmation testing and follow-up while creating related stress for families. Taking the example of PKU, a false positive test can cause a loss of about 70,000 RMB every year, not accounting the latent costs of psychological stress. So, what we should concentrate on is screening accuracy and quality management of the whole screening program in the real world.

## Conclusions

We have attempted to put forward policy suggestions for choosing diseases for MS/MS screening in Shenzhen, conducting a cost-effectiveness analysis based on the 12 IEMs recommended by NMPA. This study has confirmed that MS/MS screening covering at least three diseases is cost-effective. The cost of the MS/MS screening program detecting 12 IEMs is well within the budget constraints of the Shenzhen government. This study also discussed two policy concepts for selecting IEMs for detection. One represents the strategy of choosing the most cost-effective screening programs detecting highest number of IEMs. The other, considers the curability and affordability of the disease as the basis of healthcare decisions to screen suitable IEMs and achieves an ICER under the threshold and approaching the minimum value.

## Supplementary Information

**Additional file 1:** **S1. **Age-specific mortality and sequelae incidence of IEMs.

**Additional file 2:** **S2. **How to calculate the treatment cost of IEMs.

**Additional file 3:** **S3. **Actual rate of the medical insurance reimbursement in China.

## Data Availability

All data generated or analysed during this study can be available publicly. Readers can contact the corresponding author if there are any questions about the data or requirements for more detailed data.

## Abbreviations

BIA: Budget impact analysis
CAH: Congenital adrenal hyperplasia
CH: Congenital hypothyroidism
CIT-I: Citrullinemia type I
CIT-II: Citrullinemia type II
DBS: Dried blood spot
DD: Development Delay
GAI: Glutaric acidemia type I
G6PD deficiency: Glucose-6-phosphate Dehydrogenase deficiency
GC/MS: Gas chromatography/mass spectrometry
HCY: Homocystinuria
ICER: Incremental cost-effectiveness ratio
IEMs: Inborn errors of metabolism
IF: Immunofluorescence
IVA: Isovaleric acidemia
LC/MS: Liquid chromatography/mass spectrometry
MCAD: Medium-chain acyl-CoA dehydrogenase deficiency
MMA: Methylmalonic acidemia
MR: Mental Retardation
MS/MS: Tandem mass spectrometry
MSUD: Maple syrup urine disease
NBS: Newborn screening
ND: Neurological Damage
NMPA: National Medical Products Administration of China
PA: Propionic acidemia
PCD: Primary carnitine deficiency
PKU: Phenylalanine
QALYs: Quality-adjusted life-years
RD: Renal Damage
VLCAD: Very long-chain acyl-CoA dehydrogenase deficiency
WHO: World Health Organization
WTP: Willingness to pay
