# Supplementary material for: Cost-effectiveness analysis of newborn screening by tandem mass spectrometry in Shenzhen, China: value and affordability of new screening technology
Source: BMC Health Serv Res. 2022 Aug 15;22:1039. doi: 10.1186/s12913-022-08394-4 (PMC9376130; doi:10.1186/s12913-022-08394-4)
Supplement: Supplementary file 1 — Additional file 1: S1. Age-specific mortality and sequelae incidence of IEMs. [file 12913_2022_8394_MOESM1_ESM.docx]

**S1—A**[**ge-specific**](D:/%E7%BD%91%E9%A1%B5%E4%B8%8B%E8%BD%BD/Dict/8.9.6.0/resultui/html/index.html#/javascript:;) [**mortality**](D:/%E7%BD%91%E9%A1%B5%E4%B8%8B%E8%BD%BD/Dict/8.9.6.0/resultui/html/index.html#/javascript:;) **and sequelae incidence of IEMs**

The effectiveness of the screening program depends on whether the disease can be successfully detected in its early stages. If so, it can be followed by a series of early medical treatments to prevent life-threatening or long-term sequelae. So, we can compare the effectiveness of different screening programs as long as we know precisely the [age-specific](D:/%E7%BD%91%E9%A1%B5%E4%B8%8B%E8%BD%BD/Dict/8.9.6.0/resultui/html/index.html#/javascript:;) [mortality](D:/%E7%BD%91%E9%A1%B5%E4%B8%8B%E8%BD%BD/Dict/8.9.6.0/resultui/html/index.html#/javascript:;) of diseases and the incidence of sequelae, in both current screening and expanded screening.

Since PKU is detected in the current screening and the sensitivity of IF and MS/MS are both 100%, we assumed that the mortality and sequelae incidence of PKU are the same in the two screening programs. The other 11 diseases were not covered in the current screening, so we labeled the current screening program for the diseases as the “non-screening group” and the expanded screening as the “screening group”.

The event probabilities of CIT II, PCD and VLCAD are inaccessible, so we substituted the data of CIT I for CIT II, and MCAD for PCD and VLCAD, based on the fact that the diseases belong to the same IEM category and cause the same sequelae.

The rate of death due to other causes was added to the model as a parameter, and we extracted the data from *the* *sixth national population census of China (2010)*.

1. [**Age-specific**](D:/%E7%BD%91%E9%A1%B5%E4%B8%8B%E8%BD%BD/Dict/8.9.6.0/resultui/html/index.html#/javascript:;)  [**mortality**](D:/%E7%BD%91%E9%A1%B5%E4%B8%8B%E8%BD%BD/Dict/8.9.6.0/resultui/html/index.html#/javascript:;)

| Diseases | **Screening** | | **Non-screening** | | **Reference** |
| --- | --- | --- | --- | --- | --- |
|  | Age group | Mortality | Age group | Mortality |  |
| MSUD | 0-4 | 0.0100 | 0 | 0.7500 | [1, 2] |
|  | 5-16 | 0.0100 | 1 | 0.1500 |  |
|  | 17- | 0.0000 | 2- | 0.0000 |  |
| HCY | 0-1 | 0.0200 | 0-1 | 0.0300 | [2, 3] |
|  | 2-4 | 0.0100 | 2 | 0.0200 |  |
|  | 5-6 | 0.0070 | 3 | 0.0100 |  |
|  | 7-9 | 0.0050 | 4-5 | 0.0070 |  |
|  | 10-18 | 0.0001 | 6-8 | 0.0050 |  |
|  | 19- | 0.0000 | 9-18 | 0.0001 |  |
|  |  |  | 19- | 0.0000 |  |
| CIT I、II | 0 | 0.2000 | 0 | 0.3000 | [2] |
|  | 1-3 | 0.1000 | 1 | 0.2000 |  |
|  | 4 | 0.0500 | 2-3 | 0.1000 |  |
|  | 5 | 0.0200 | 4 | 0.0500 |  |
|  | 6-7 | 0.0100 | 5 | 0.0200 |  |
|  | 8-10 | 0.0050 | 6-7 | 0.0100 |  |
|  | 11- | 0.0000 | 8-10 | 0.0050 |  |
|  |  |  | 11- | 0.0000 |  |
| GA I | 0-3 | 0.0100 | 0-15 | 0.0200 | [2, 4] |
|  | 4-7 | 0.0025 | 16- | 0.0000 |  |
|  | 8-15 | 0.0001 |  |  |  |
|  | 16- | 0.0000 |  |  |  |
| IVA、MMA、  PA | 0 | 0.0400 | 0-1 | 0.2000 | [2, 5] |
|  | 1 | 0.0200 | 2 | 0.0500 |  |
|  | 2-3 | 0.0100 | 3-6 | 0.0100 |  |
|  | 4-9 | 0.0050 | 7-9 | 0.0050 |  |
|  | 10- | 0.0000 | 10- | 0.0000 |  |
| MCAD、PCD、  VLCAD | 0-2 | 0.0100 | 0 | 0.1200 | [2, 6] |
|  | 3 | 0.0050 | 1 | 0.0400 |  |
|  | 4-7 | 0.0010 | 2-10 | 0.0100 |  |
|  | 8-32 | 0.0001 | 11- | 0.0000 |  |
|  | 33- | 0.0000 |  |  |  |
| PKU | 0-9 | 0.00253 |  |  | [7] |
|  | 10-19 | 0.0288 |  |  |  |
|  | 20-29 | 0.00457 |  |  |  |
|  | 30-39 | 0.00439 |  |  |  |
|  | 40-49 | 0.00354 |  |  |  |
|  | 50-59 | 0.00324 |  |  |  |
|  | 60- | 0.01130 |  |  |  |

1. **Sequelae incidence**

| Diseases | Screening | | Non-screening | | Reference |
| --- | --- | --- | --- | --- | --- |
|  | Age group | Mortality | Age group | Mortality |  |
| MSUD | | | | | [1, 2] |
| DD | 0 | 0.01 | 0 | 0.010 |  |
|  | 1 | 0.005 | 1-4 | 0.010 |  |
|  | 2-8 | 0.001 | 5-16 | 0.001 |  |
|  | 9- | 0.000 | 17- | 0.000 |  |
| ND | 0 | 0.02 | 0 | 0.020 |  |
|  | 1-3 | 0.02 | 1-3 | 0.020 |  |
|  | 4 | 0.01 | 4 | 0.01 |  |
|  | 5-16 | 0.001 | 5-16 | 0.001 |  |
|  | 17- | 0.000 | 17- | 0.000 |  |
| HCY | | | | | [2, 3] |
| MR | 0 | 0.04 | 0 | 0.36 |  |
|  | 1 | 0.020 | 1 | 0.225 |  |
|  | 2-3 | 0.010 | 2 | 0.090 |  |
|  | 4 | 0.005 | 3 | 0.045 |  |
|  | 5-9 | 0.001 | 4 | 0.036 |  |
|  | 10- | 0.000 | 5-14 | 0.007 |  |
|  |  |  | 15- | 0.001 |  |
| CIT I、CIT II | | | | | [2] |
| MR | 0 | 0.040 | 0 | 0.040 |  |
|  | 1-4 | 0.040 | 1-4 | 0.040 |  |
|  | 5- | 0.000 | 5- | 0.000 |  |
| GA I | | | | | [2, 4] |
| ND | 0 | 0.050 | 0 | 0.350 |  |
|  | 1-4 | 0.050 | 1 | 0.270 |  |
|  | 5 | 0.040 | 2 | 0.120 |  |
|  | 6-7 | 0.020 | 3 | 0.060 |  |
|  | 8 | 0.010 | 4 | 0.040 |  |
|  | 9-10 | 0.005 | 5 | 0.030 |  |
|  | 11-15 | 0.0001 | 6-7 | 0.010 |  |
|  | 16- | 0.000 | 8-10 | 0.005 |  |
|  |  |  | 11-15 | 0.0001 |  |
|  |  |  | 16- | 0.000 |  |
| IVA、MMA、PA | | | | | [2, 5] |
| ND | 0 | 0.006 | 0 | 0.009 |  |
|  | 1 | 0.006 | 1 | 0.009 |  |
|  | 2-4 | 0.012 | 2-9 | 0.045 |  |
|  | 5-9 | 0.015 | 10-14 | 0.071 |  |
|  | 10-14 | 0.030 | 15- | 0.000 |  |
|  | 15- | 0.000 |  |  |  |
| RD | 0 | 0.000 | 0 | 0.001 |  |
|  | 1-5 | 0.000 | 1-3 | 0.001 |  |
|  | 6-10 | 0.010 | 4-5 | 0.002 |  |
|  | 11- | 0.000 | 6-10 | 0.06 |  |
|  |  |  | 11-12 | 0.05 |  |
|  |  |  | 13-14 | 0.002 |  |
|  |  |  | 15- | 0.000 |  |
| MCAD、PCD、VLCAD | | | | | [2, 6] |
| DD | 0 | 0.040 | 0 | 0.080 |  |
|  | 1- | 0.040 | 1- | 0.040 |  |
| PKU | | | | | [7-9] |
| ND | 0 | 0.134 | 0 | 0.670 |  |
|  | 1- | 0.134 | 1- | 0.154 |  |

1. **Probability of death due to other causes[10]：**

| **Age group** | **Mortality（‰）** | **Age group** | **Mortality（‰）** |
| --- | --- | --- | --- |
| 0 | 3.82 | 35-39 | 1.16 |
| 1 | 1.11 | 40-44 | 1.76 |
| 2 | 0.63 | 45-49 | 2.61 |
| 3 | 0.45 | 50-54 | 4.18 |
| 4 | 0.37 | 55-59 | 6.19 |
| 5-9 | 0.3 | 60-64 | 10.31 |
| 10-14 | 0.3 | 65-69 | 17.21 |
| 15-19 | 0.39 | 70-74 | 30.64 |
| 20-24 | 0.5 | 75-79 | 49.52 |
| 25-29 | 0.61 | 80-84 | 84.81 |
| 30-34 | 0.81 |  |  |

**Reference：**

1. Morton, D.H., et al., *Diagnosis and treatment of maple syrup disease: a study of 36 patients.* Pediatrics, 2002. **109**(6): p. 999-1008.

2. Tiwana, S.K., *Expanded Newborn Screening in Texas: A Cost-effectiveness Analysis Using Markov Modeling*. 2009, The University of Texas at Austin: Ann Arbor. p. 198.

3. Mudd, S.H., et al., *The natural history of homocystinuria due to cystathionine beta-synthase deficiency.* Am J Hum Genet, 1985. **37**(1): p. 1-31.

4. Strauss, K.A., et al., *Type I glutaric aciduria, part 1: natural history of 77 patients.* Am J Med Genet C Semin Med Genet, 2003. **121c**(1): p. 38-52.

5. Dionisi-Vici, C., et al., *'Classical' organic acidurias, propionic aciduria, methylmalonic aciduria and isovaleric aciduria: long-term outcome and effects of expanded newborn screening using tandem mass spectrometry.* J Inherit Metab Dis, 2006. **29**(2-3): p. 383-9.

6. Wilcken, B., J. Hammond, and M. Silink, *Morbidity and mortality in medium chain acyl coenzyme A dehydrogenase deficiency.* Arch Dis Child, 1994. **70**(5): p. 410-2.

7. Thiboonboon, K., et al., *An Economic Evaluation of Neonatal Screening for Inborn Errors of Metabolism Using Tandem Mass Spectrometry in Thailand.* PLoS One, 2015. **10**(8): p. e0134782.

8. Scriver, C.R., et al., *The Metabolic and Molecular Bases of Inherited Disease*. Vol. 8th Edition. 2001, New-York: McGraw-Hill.

9. González, M.J., et al., *Neurological complications and behavioral problems in patients with phenylketonuria in a follow-up unit.* Mol Genet Metab, 2011. **104 Suppl**: p. S73-9.

10. National Bereau of Statistics. *[The sixth national population census of China]*. 2012 [cited 2021; Available from: <http://www.stats.gov.cn/tjsj/pcsj/rkpc/6rp/indexch.htm>.
