# Supplementary material for: Cost-effectiveness analysis of newborn screening by tandem mass spectrometry in Shenzhen, China: value and affordability of new screening technology
Source: BMC Health Serv Res. 2022 Aug 15;22:1039. doi: 10.1186/s12913-022-08394-4 (PMC9376130; doi:10.1186/s12913-022-08394-4)
Supplement: Supplementary file 2 — Additional file 2: S2. How to calculate the treatment cost of IEMs. [file 12913_2022_8394_MOESM2_ESM.docx]

**S2—How to calculate the treatment cost of IEMs**

Schoen et al. [1] divided the cost of treatment into two parts at the 5 years of age because, in some cases, additional care is needed for children with IEMs detected after symptoms manifest during their first five years. We also found discrepancies between different ages in the treatment and medication criteria for IEMs when referring to *Guidelines for the Treatment of Rare Diseases (2019)* [2]. So, we did the same to estimate the treatment cost, dividing patients into two groups at age 5.

The treatment cost of IEMs mainly includes the cost of special infant formulas and primary medications. If the treatment and medication criteria for IEMs are introduced in detail in the guidelines, then the treatment cost can be estimated by adding the cost of each additional component; otherwise, we will refer to other studies.

The following [assumption](D:/%E7%BD%91%E9%A1%B5%E4%B8%8B%E8%BD%BD/Dict/8.9.6.0/resultui/html/index.html#/javascript:;)s and calculation bases are used:

1. An infant can drink 1800g milk formulas around every month [3], and patients should drink this over their lifetime to ensure normal physical growth and development of physiological functions. The price of special formulas is derived from a propriety limited food science and technology company in Shanghai.
2. For drug prices, we referred to an online pharmacy, and chose the recommended retail price of the drug sold in most pharmacies [4, 5]. Patients should take the drugs for life and adhere to prescribed dosages.
3. The weight of patients younger than 6 years old (but not including the age of 6) is calculated at 15kg and the other is 50kg.
4. The estimated treatment cost may be lower than the truth-value, since we did not take other costs, like the cost of the [examination](D:/%E7%BD%91%E9%A1%B5%E4%B8%8B%E8%BD%BD/Dict/8.9.6.0/resultui/html/index.html#/javascript:;),[emergency treatment](D:/%E7%BD%91%E9%A1%B5%E4%B8%8B%E8%BD%BD/Dict/8.9.6.0/resultui/html/index.html#/javascript:;) and so on, into account. Therefore, the final cost of treatment was multiplied by 1.1 as an [adjustment](D:/%E7%BD%91%E9%A1%B5%E4%B8%8B%E8%BD%BD/Dict/8.9.6.0/resultui/html/index.html#/javascript:;) [coefficient](D:/%E7%BD%91%E9%A1%B5%E4%B8%8B%E8%BD%BD/Dict/8.9.6.0/resultui/html/index.html#/javascript:;).

| **Diseases** | **Treatment** | **0-5 years old** | | **6-years old** | | **Total cost per year** | |
| --- | --- | --- | --- | --- | --- | --- | --- |
|  |  | [**Dosage**](D:/%E7%BD%91%E9%A1%B5%E4%B8%8B%E8%BD%BD/Dict/8.9.6.0/resultui/html/index.html#/javascript:;) | **Cost per year** | [**Dosage**](D:/%E7%BD%91%E9%A1%B5%E4%B8%8B%E8%BD%BD/Dict/8.9.6.0/resultui/html/index.html#/javascript:;) | **Cost per year** | **0-5**  **years old** | **6-**  **years old** |
| MSUD | milk formulas/can ^a^ | 5 per month | 12000 | 2 per month | 4800 | 13440.90 | 6083.00 |
|  | VitB1/g ^b^ | 10 mg/（kg·d） | 219.00 | 10 mg/（kg·d） | 730.00 |  |  |
| HCY | milk formulas/can | 5 per month | 12000 | 2 per month | 4800 | 13772.13 | 5952.51 |
|  | VitB6/g ^c^ | 250 mg/d | 465.37 | 250 mg/d | 465.37 |  |  |
|  | [glycine](D:/%E7%BD%91%E9%A1%B5%E4%B8%8B%E8%BD%BD/Dict/8.9.6.0/resultui/html/index.html#/javascript:;) [betaine](D:/%E7%BD%91%E9%A1%B5%E4%B8%8B%E8%BD%BD/Dict/8.9.6.0/resultui/html/index.html#/javascript:;)/g ^d^ | 200mg/（kg·d) | 54.75 | 8 g/d | 146.00 |  |  |
| IVA | milk formulas/can | 5 per month | 12000 | 2 per month | 4800 | 17567.81 | 13045.01 |
|  | [Levocarnitine](D:/%E7%BD%91%E9%A1%B5%E4%B8%8B%E8%BD%BD/Dict/8.9.6.0/resultui/html/index.html#/javascript:;)/15g ^e^ | 75m g/（kg·d） | 3970.74 | 40mg/（kg·d） | 7059.10 |  |  |
| GA I | milk formulas/can | 5 per month | 12000 | 2 per month | 4800 | 17567.81 | 13045.01 |
|  | [Levocarnitine](D:/%E7%BD%91%E9%A1%B5%E4%B8%8B%E8%BD%BD/Dict/8.9.6.0/resultui/html/index.html#/javascript:;)/15g | 75mg/（kg·d） | 3970.74 | 40mg/（kg·d） | 7059.1 |  |  |
| MMA | milk formulas/can | 5 per month | 12000 | 2 per month | 4800 | 19023.76 | 24692.53 |
|  | [Levocarnitine](D:/%E7%BD%91%E9%A1%B5%E4%B8%8B%E8%BD%BD/Dict/8.9.6.0/resultui/html/index.html#/javascript:;)/15g | 100mg/（kg·d） | 5294.33 | 100mg/（kg·d） | 17647.75 |  |  |
| PA | milk formulas/can | 5 per month | 12000 | 2 per month | 4800 | 17567.81 | 19839.39 |
|  | [Levocarnitine](D:/%E7%BD%91%E9%A1%B5%E4%B8%8B%E8%BD%BD/Dict/8.9.6.0/resultui/html/index.html#/javascript:;)/15g | 75mg/（kg·d） | 3970.74 | 75mg/（kg·d） | 13235.81 |  |  |
| CIT I, CIT II | CIT I, CIT II and HCY are all metabolic disorders of amino acids and the sequelae are the same, so we believe that we can estimate the treatment cost of CIT I and CIT II using the treatment cost of HCY. According to the published article, the treatment cost of HCY is 3.5 times higher than for CIT I and CIT II [6]. | | | | | 48202.46 | 20833.77 |
| MCAD, PCD, VLCAD | MCAD, PCD and VLCAD are all metabolic disorders of fatty acids and the sequelae are the same, so we assumed that the treatment cost of the three diseases is also the same. The study of [Marion Haas](https://pubmed.ncbi.nlm.nih.gov/?size=50&term=Haas+M&cauthor_id=17643760) et al. showed that the treatment cost of MCAD was A$1796 (in 2007) for children who were clinically diagnosed [7]. We then converted A$ into RMB using the exchange rate of 6.3768 and discounted the cost to 2018 at an annual rate of 3%. | | | | | 15853.26 | 15853.26 |

* The cost is presented in 2018 RMB.

A． The prices of milk formulas for different IEMs are all 200 RMB per can (400g).

B． The price of VitB1 is 4.00 RMB for 10mg*100 pieces. Access: <https://www.315jiage.cn/mn90858.aspx.> Date: March. 2021.

C． The price of VitB6 is 5.10 RMB for 10mg*100 pieces. Access: <https://www.315jiage.cn/mn90873.aspx.> Date: March. 2021.

D．The price of [glycine](D:/%E7%BD%91%E9%A1%B5%E4%B8%8B%E8%BD%BD/Dict/8.9.6.0/resultui/html/index.html#/javascript:;) [betaine](D:/%E7%BD%91%E9%A1%B5%E4%B8%8B%E8%BD%BD/Dict/8.9.6.0/resultui/html/index.html#/javascript:;) is 50 RMB/kg. Access: https://china.guidechem.com/trade/pdetail21199937.html. Date: March. 2021.

E． The price of [Levocarnitine](D:/%E7%BD%91%E9%A1%B5%E4%B8%8B%E8%BD%BD/Dict/8.9.6.0/resultui/html/index.html#/javascript:;) is 58.02 RMB for 1g*6 pieces. Access: https://www.315jiage.cn/mn87411.aspx. Date: March. 2021.

**Reference：**

1. Schoen, E.J., et al., *Cost-benefit analysis of universal tandem mass spectrometry for newborn screening.* Pediatrics, 2002. **110**(4): p. 781-6.

2. National Health Commission of the People’s Republic of China. *[Guidelines for the Treatment of Rare Diseases (2019)]*. 2019 [cited 2021; Available from: <http://www.nhc.gov.cn/yzygj/s7659/201902/61d06b4916c348e0810ce1fceb844333.shtml>.

3. Yao, X. and W. Shen, *Bei Jing Shi Ying You Er Nai Fen Shi Chang Diao Yan [Analysis of infant milk powder market in Beijing].* Journal of Beijing University of Agriculture, 2012. **27**(03): p. 49-52.

4. Drug Price 315-Website. *[Drug Prices Enquiry]*. 2021 [cited 2021; Available from: <https://www.315jiage.cn/>.

5. Usolf. *[The price of trimethylglycine]*. 2021 [cited 2021; Available from: <https://detail.1688.com/offer/547752451154.html?spm=a261b.12436309.ul20190116.86.6efa360041rofX>.

6. Tiwana, S.K., K.L. Rascati, and H. Park, *Cost-effectiveness of expanded newborn screening in Texas.* Value Health, 2012. **15**(5): p. 613-21.

7. Haas, M., et al., *Healthcare use and costs of medium-chain acyl-CoA dehydrogenase deficiency in Australia: screening versus no screening.* J Pediatr, 2007. **151**(2): p. 121-6, 126.e1.
