# Supplementary material for: Cost-effectiveness analysis of newborn screening by tandem mass spectrometry in Shenzhen, China: value and affordability of new screening technology
Source: BMC Health Serv Res. 2022 Aug 15;22:1039. doi: 10.1186/s12913-022-08394-4 (PMC9376130; doi:10.1186/s12913-022-08394-4)
Supplement: Supplementary file 3 — Additional file 3: S3. Actual rate of the medical insurance reimbursement in China. [file 12913_2022_8394_MOESM3_ESM.docx]

**S3—Actual rate of the medical insurance reimbursement in China**

Previously, the Chinese basic medical security system comprised of three major insurance schemes, namely, the Urban Employee Basic Medical Insurance (UEBMI), the Urban Resident Basic Medical Insurance (URBMI), and the New Cooperative Medical Scheme (NCMS). Later, URBMI and NCMS were combined in the Urban and Rural Residents Basic Medical Insurance (URRBMI).

According to the *Statistical Bulletin on National Medical Insurance Development* released in 2019 by the National Healthcare Security Administration of China [1], the actual rate of UEBMI reimbursement for hospitalization expenses in 2019 was 75.6% and URRBMI was 59.7%. For outpatient expenses, the rates were 42% and 36% respectively, referring to the *CHIRA-DATA blue book* (a grey document jointly published by IQVIA and Chinese Organization for Rare Disorders, which is not available online)*.*

Considering the complexity of treatments for IEMs, we chose the representative ratio of hospitalization expenses and outpatient expenses in tertiary hospitals, with the help of *China Health and Healthcare Statistical Yearbook (2019)* [2]. The ratio was 2:1. So, based on the data above:

((75.6%*(2/3) +42%*(1/3)) +(59.7%*(2/3)+36%*(1/3)))*(1/2)=58.10%

We estimated the actual rate of medical insurance reimbursement in China to be around 60%.

**Reference：**

1. National Healthcare Security Administration. *[Statistical Bulletin on National Medical Insurance Development]*. 2020 [cited 2021; Available from: <http://www.nhsa.gov.cn/art/2020/6/24/art_7_3268.html>.

2. National Health Commission of the People’s Republic of China. *[China Health and Healthcare Statistical Yearbook (2019)]*. 2020 [cited 2021; Available from: <https://data.cnki.net/yearbook/Single/N2020020200>.
